# Supplementary material for: Red Meat Intake, Indole-3-Acetate, and Dorea longicatena Together Affect Insulin Resistance after Gastric Bypass
Source: Nutrients. 2023 Feb 27;15(5):1185. doi: 10.3390/nu15051185 (PMC10005351; doi:10.3390/nu15051185)
Supplement: Supplementary file 1 [file nutrients-15-01185-s001.zip › Review Translation Certificate - Ana Prudencio.pdf]

Cardinal
The American English

Translation Certificate – February 9, 2023

I, Professor Dr. Karin Romano Posegger, as a native American, fluent in the Brazilian-Portuguese language, hereby certify that the following manuscript “Red meat intake, indole-3-acetate, and Dorea longicatena together affect insulin resistance after gastric bypass” was properly translated and reviewed. The final revision was made after the modifications suggested by the reviewers were completed by the authors of the manuscript. To the best of my knowledge, the translation from Portuguese (Brazil) language into American English language holds true and is accurate according to the grammar and spelling language requirements and rules.

Karin Romano Posegger
CPF 162.926.638-84

Prof Dr Karin R. Posegger, Ph.D., MSc.
Norsk Cardinal LLC, American English Translation and Review
Brazil: + 55 (11) 98432-3133
USA: + 1 (407) 308-6256
