# Supplementary material for: Red Meat Intake, Indole-3-Acetate, and Dorea longicatena Together Affect Insulin Resistance after Gastric Bypass
Source: Nutrients. 2023 Feb 27;15(5):1185. doi: 10.3390/nu15051185 (PMC10005351; doi:10.3390/nu15051185)
Supplement: Supplementary file 1 [file nutrients-15-01185-s001.zip › SUPPLEMETARYFILES CLEAN.pdf]

nutrients

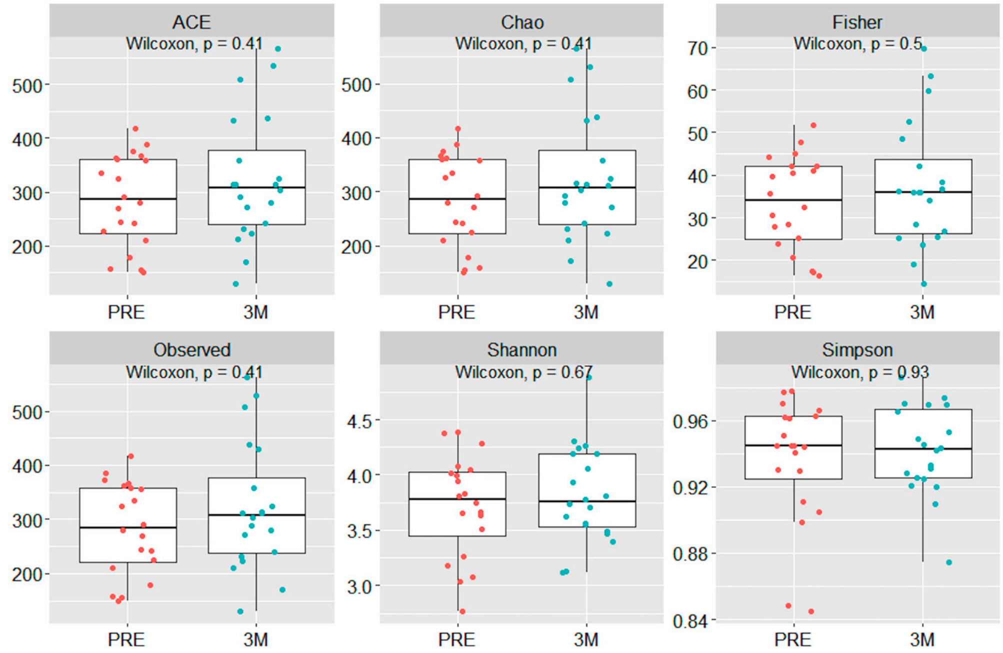

Supplementary Figure S1. Alpha diversity indices before and three months after RYGB. Statistical significance p < 0.05.

nutrients MDPI

Supplementary Table S1 - Regression models for effects of variation (Δ postoperative – preoperative) of food intake, tryptophan metabolites and gut microbiota (independent variables) on postoperative Glycemia, HbA1c, HOMA-IR and HOMA-Beta (dependent variables).

**Food intake**	**Glycemia**		**HbA1c**		**HOMA-IR**		**HOMA-Beta**	
**Model 1**	**β (95% CI)**	**p**	**β (95% CI)**	**p**	**β (95% CI)**	**p**	**β (95% CI)**	**p**
Protein (g)	-0.02 (-0.98;0.93)	0.97	0.01(-0.01, 0.03)	0.57	-0.01(-0.07, 0.04)	0.69	-1.02 (-2.98, 0.95)	0.41
Tryptophan (mg)	-0.02 (-0.10;0.06)	0.74	0.0002(-0.001, 0.002)	0.88	0.001(-0.004, 0.01)	0.85	0.02 (-0.16, 0.20)	0.86
Fiber (g)	1.28 (-0.47;3.03)	0.25	-0.01 (-0.04, 0.03)	0.77	0.03(-0.08, 0.14)	0.64	-0.25 (-4.45, 3.95)	0.93
Red meat (g)	0.10 (0.03;0.17)	0.03**	0.0002(-0.001, 0.002)	0.81	0.01(0.005, 0.01)	0.002***	-0.03 (-0.20, 0.13)	0.75
Refined cereals (g)	0.001 (-0.03; 0.03)	0.96	-0.0002 (-0.001, 0.0005)	0.60	0.0004(-0.001, 0.002)	0.72	-0.004 (-0.07, 0.07)	0.94
**Model 2**								
Protein (g)	-0.17 (1.23;0.89)	0.80	0.005 (-0.02, 0.03)	0.79	-0.05(-0.09, -0.02)	0.03**	-1.72 (-4.60, 1.17)	0.35
Tryptophan (mg)	-0.05 (-0.13;0.04)	0.39	0.0001 (-0.002, 0.002)	0.93			0.10 (-0.17, 0.37)	0.57
Fiber (g)	1.53 (-0.48;3.53)	0.24	-0.004(-0.06, 0.05)	0.90			-2.71(-9.35, 3.94)	0.52
Red meat (g)	0.10 (0.03;0.18)	0.05**	0.0001(-0.002, 0.002)	0.96	0.01 (0.01, 0.01)	0.0001***	0.03 (-0.18, 0.23)	0.85
Refined cereals (g)	-0.003 (-0.03; 0.03)	0.88	-0.0002(-0.001, 0.001)	0.72			-0.01 (-0.09, 0.07)	0.81
**Metabolites**								
**Model 1**								
Tryptophan	-0.001 (-0.002, 0.0002)	0.19	0.0000(-0.0000, 0.0000)	0.92	-0.0000(-0.0001, 0.0000)	0.52	0.002 (0.0003, 0.004)	0.09*
N-acetyl-serotonin	-0.0000 (-0.001, 0.001)	0.94	0.0000(-0.0000, 0.0000)	0.35	-0.0000(-0.0001, 0.0000)	0.27	-0.001(-0.002, 0.001)	0.32
Indole-3-acetate	0.003 (-0.01, 0.01)	0.58	0.0001(-0.0001, 0.0003)	0.38	-0.001(-0.002, 0.0002)	0.21	-0.01(-0.05, 0.02)	0.57
Glutamic acid	-0.002 (-0.01, 0.003)	0.49	-0.0001(-0.0002, 0.0000)	0.32	0.0002(-0.0001, 0.001)	0.34	0.01(-0.002, 0.02)	0.18
L-alanine	0.0001 (-0.0002, 0.0004)	0.52	-0.0000(-0.0000, 0.0000)	0.15	0.0000(-0.0000, 0.0000)	0.28	0.0001 (-0.0005, 0.001)	0.75
α-ketoglutarate acid	-0.01 (-0.04, 0.03)	0.69	-0.0004(-0.001, 0.0002)	0.28	-0.0004(-0.002, 0.002)	0.78	-0.01 (-0.09, 0.06)	0.77
Benzoic acid	-0.004 (-0.01, 0.003)	0.39	-0.0002(-0.0003, -0.0001)	0.04**	-0.0002(-0.001, 0.0002)	0.35	0.001 (-0.01, 0.02)	0.91
Anthranilic acid	0.002 (-0.001, 0.01)	0.22	-0.0000(-0.0001, 0.0000)	0.61	0.0002 (0.0000, 0.0004)	0.10*	0.001 (-0.01, 0.01)	0.76

Continue on the next page

**Metabolites**	**Glycemia**		**HbA1c**		**HOMA-IR**		**HOMA-Beta**	
**Model 2**	**β (95% CI)**	**p**	**β (95% CI)**	**p**	**β (95% CI)**	**p**	**β (95% CI)**	**p**
Tryptophan	-0.001 (-0.002, -0.0004)	0.06*	0.0000 (-0.0000, 0.0000)	0.52	-0.0000 (-0.0001, 0.0001)	0.63	0.003 (-0.0004, 0.01)	0.20
N-acetyl-serotonin	0.001 (-0.0003, 0.001)	0.32	-0.0000 (-0.0000, 0.0000)	0.81	-0.0000 (-0.0001, 0.0000)	0.81	-0.001 (-0.003, 0.001)	0.41
Indole-3-acetate			0.0002 (-0.0001, 0.0004)	0.28	-0.001 (-0.002, 0.0004)	0.34	0.01 (-0.03, 0.05)	0.74
Glutamic acid	0.01 (-0.002, 0.02)	0.22	-0.0000 (-0.0002, 0.0001)	0.62	0.0003 (-0.0003, 0.001)	0.48	0.01 (-0.01, 0.03)	0.58
L-alanine	0.0003 (0.0000, 0.001)	0.13	-0.0000 (-0.0000, 0.0000)	0.35	0.0000 (-0.0000, 0.0000)	0.52	-0.0001 (-0.001, 0.001)	0.81
α-ketoglutarate acid			-0.0002 (-0.001, 0.001)	0.70	-0.001 (-0.004, 0.002)	0.69	-0.05 (-0.17, 0.07)	0.55
Benzoic acid	-0.01 (-0.01, 0.0003)	0.17	-0.0001(-0.0003, 0.0001)	0.37	-0.0004 (-0.001, 0.0002)	0.29	-0.002 (-0.02, 0.02)	0.90
Anthranilic acid			0.0000 (-0.0001, 0.0001)	0.61	0.0001 (-0.0002, 0.0004)	0.74	0.004 (-0.01, 0.02)	0.61
**Gut Microbiota**								
**Model 1**								
Akkermansia muciniphila (sq15)	-0.0003 (-0.001, 0.001)	0.58	-0.0000 (-0.0000, 0.0000)	0.40	0.0000 (-0.0000, 0.0001)	0.49	0.001 (-0.001, 0.003)	0.55
Clostridium tarantellae (sq338)	-0.004 (-0.04, 0.03)	0.86	-0.0002 (-0.001, 0.0005)	0.63	-0.001 (-0.002, 0.001)	0.57	-0.05 (-0.12, 0.01)	0.20
Fusobacterium nucleatum (sq381)	0.09 (0.02, 0.16)	0.05**	0.001 (-0.0004, 0.003)	0.23	0.002 (-0.01, 0.01)	0.68	0.18 (-0.17, 0.53)	0.41
Fusobacterium periodonticum (sq368)	-0.004 (-0.05, 0.04)	0.91	0.0001 (-0.001, 0.001)	0.87	-0.001 (-0.003, 0.002)	0.63	-0.02 (-0.12, 0.08)	0.70
Gemella haemolysans (sq311)	-0.01 (-0.05, 0.03)	0.67	0.0004 (-0.0005, 0.001)	0.48	-0.001 (-0.003, 0.002)	0.56	0.04 (-0.06, 0.13)	0.52
Streptococcus salivarius (sq28)	0.001 (-0.0005, 0.003)	0.25	0.0000 (0.0000, 0.0001)	0.12	-0.0001 (-0.0004, 0.0002)	0.51	-0.002 (-0.01, 0.01)	0.80
Blautia luti (sq96)	0.001 (-0.01, 0.01)	0.94	0.0000 (-0.0002, 0.0003)	0.77	-0.0001 (-0.001, 0.001)	0.80	-0.001 (-0.03, 0.02)	0.98

Continue on the next page

**Gut Microbiota**	**Glycemia**		**HbA1c**		**HOMA-IR**		**HOMA-Beta**	
**Model 1**	**β (95% CI)**	**p**	**β (95% CI)**	**p**	**β (95% CI)**	**p**	**β (95% CI)**	**p**
Clostridium clostridioforme (sq691)	-0.1 (-0.4, 0.2)	0.60	-0.01 (-0.01, -0.000)	0.20	-0.01 (-0.02, 0.01)	0.40	-0.4 (-0.9, 0.2)	0.40
Clostridium lavalense (sq380)	0.01 (-0.05, 0.1)	0.90	0.001 (-0.001, 0.002)	0.50	-0.001 (-0.004, 0.002)	0.70	-0.1(-0.2, 0.04)	0.40
Dialister invisus (sq1316)	0.4 (-1.3, 2.1)	0.70	-0.01 (-0.1, 0.02)	0.60	-0.1 (-0.2, 0.04)	0.40	-1.4 (-5.9, 3.1)	0.70
Dorea longicatena (sq1408)	0.9 (0.1, 1.7)	0.10*	0.003 (-0.01, 0.02)	0.80	0.1 (0.01, 0.1)	0.10*	-1.9 (-3.6, -0.1)	0.10*
Eisenbergiella tayi (sq768)	-0.1 (-0.2, 0.1)	0.50	-0.001 (-0.004, 0.002)	0.70	-0.002 (-0.01, 0.01)	0.70	0.02 (-0.3, 0.3)	1.00
Enterococcus faecalis (sq1127)	-0.05 (-0.7, 0.6)	1.00	-0.01(-0.02, 0.01)	0.40	-0.01(-0.05, 0.03)	0.80	-0.8 (-2.2, 0.6)	0.40
Escherichia coli (sq3)	-0.000 (-0.001, 0.000)	0.60	-0.000(-0.000, 0.000)	0.60	-0.000(-0.000, 0.000)	0.90	0.000 (-0.001, 0.001)	1.00
Faecalibacterium prausnitzii (sq437)	0.05 (-0.1, 0.2)	0.60	0.000(-0.003, 0.003)	1.00	-0.001(-0.01, 0.01)	0.80	-0.1(-0.4, 0.2)	0.70
Faecalibacterium prausnitzii (sq649)	0.1 (-0.1, 0.3)	0.50	0.002(-0.002, 0.01)	0.40	-0.01(-0.02, 0.01)	0.70	0.4 (-0.3, 1.1)	0.40
Fusobacterium nucleatum (sq1129)	0.5 (-0.1, 1.1)	0.30	0.005(-0.01, 0.02)	0.60	-0.01(-0.1, 0.04)	0.90	0.4(-1.5, 2.4)	0.80
Fusobacterium nucleatum (sq612)	0.01 (-0.14, 0.16)	0.95	0.001(-0.003, 0.004)	0.78	-0.004(-0.01, 0.004)	0.42	-0.11 (-0.43, 0.22)	0.60
Granulicatella adiacens (sq421)	-0.01 (-0.07, 0.05)	0.77	0.001(-0.001, 0.002)	0.44	-0.002(-0.01, 0.002)	0.47	0.04(-0.10, 0.18)	0.63
Mogibacterium vescum (sq1698)	-0.56 (-2.64, 1.52)	0.67	-0.01(-0.06, 0.03)	0.63	-0.002(-0.19, 0.18)	0.99	-2.04 (-8.96, 4.88)	0.64
Neisseria flavescens (sq647)	-0.02 (-0.32, 0.27)	0.90	-0.005(-0.01, 0.001)	0.19	-0.01(-0.03, 0.01)	0.32	-0.26(-0.87, 0.36)	0.50
Oribacterium parvum (sq1542)	-0.24 (-1.19, 0.72)	0.69	0.01 (-0.01, 0.03)	0.53	-0.02(-0.07, 0.04)	0.60	0.71 (-1.33, 2.75)	0.58
Streptococcus parasanguinis (sq93)	0.005 (-0.002, 0.01)	0.28	0.0001 (0.0000, 0.0003)	0.11	-0.0004 (-0.001, 0.0005)	0.49	-0.003 (-0.04, 0.03)	0.89

*p≤0.1; **p≤0.05; ***p≤0.01. Food intake: Model 1 – univariate regression; Model 2 – multivariable regression. Metabolites: Model 1 – univariate regression; Model 2 – multivariable regression: HOMA-IR*** R^2 0.68, Adjusted R^2 0.63. Gut microbiota: Model 1 – univariate regression. Statistical significance $p < 0.05$.
